# Mechanisms of acupuncture in the treatment of type 2 diabetes mellitus: insights from the regulation of the gut microecology

**DOI:** 10.3389/fimmu.2025.1682521

**Published:** 2025-11-20

**Authors:** Huifang Niu, Minfeng Zhou, Li Zhou, Huan Wu, Fan Wu, Shuxiu Zhu, Rui Chen, Fengxia Liang, Hongxing Zhang

**Affiliations:** 1 Medical School, Jianghan University, Wuhan, Hubei, China; 2 Institute of Acupuncture, Jianghan University, Wuhan, Hubei, China; 3 Department of Integrated Traditional Chinese and Western Medicine, Union Hospital Affiliated to Tongji Medical College, Huazhong University of Science and Technology, Wuhan, Hubei, China; 4 School of Acupuncture and Bone Injury, Hubei University of Chinese Medicine, Wuhan, Hubei, China

**Keywords:** acupuncture therapy, type 2 diabetes mellitus, gut microecology, gut microbiota, insulin signaling pathway

## Abstract

Type 2 diabetes mellitus (T2DM) is a chronic metabolic disorder with a complex etiology, in which gut microecological dysregulation plays a central role in disease onset and progression. Acupuncture, a non-pharmacological intervention rooted in traditional Chinese medicine, confers multi-target and systemic regulatory effects, and has been extensively applied in the prevention and management of T2DM. This review systematically examines the roles of gut microbiota dysbiosis, aberrant gut-derived metabolites, intestinal barrier dysfunction, and chronic low-grade inflammation in the pathophysiology of T2DM. It further delineates the potential mechanisms by which acupuncture indirectly mitigates T2DM through modulation of the “gut microbiota–metabolite–intestinal barrier–inflammation” axis, alongside its direct actions on insulin signaling pathways and pancreatic β-cell function. Drawing on recent advances in multi-omics technologies, this review discusses the prospects and challenges of applying acupuncture in individualized and precision treatment of T2DM, aiming to provide a conceptual framework and theoretical foundation for an integrated diabetes prevention and treatment paradigm with distinctive features of traditional Chinese medicine.

## 1 Introduction

Type 2 diabetes mellitus (T2DM) is a chronic metabolic disorder characterized by insulin resistance and progressive β-cell dysfunction. With a steadily rising prevalence, it has emerged as a major global public health concern (1, 2). According to the 10th edition of the International Diabetes Federation Diabetes Atlas, an estimated 537 million people worldwide were living with diabetes in 2021, a figure projected to reach 783 million by 2045 (3). The onset and progression of T2DM impose a substantial burden on patient quality of life and socioeconomic systems (4). Although current hypoglycemic agents achieve variable glycemic control, their efficacy is often limited by pronounced inter-individual variability, transient effects, and a spectrum of adverse reactions coupled with significant economic costs. These factors severely undermine long-term patient adherence and constrain widespread clinical adoption (5). Consequently, the development of more effective, safe, and sustainable intervention strategies has emerged as a critical focus in T2DM research.

Compared to the non-specific side effects of pharmacological treatments, acupuncture is characterized by multi-pathway, multi-target regulation and a favorable safety profile. It holds distinct advantages in managing metabolic disorders, particularly through modulating gut microenvironment homeostasis, enhancing insulin sensitivity, and promoting insulin secretion (6–8). Recent preclinical and clinical studies have demonstrated that acupuncture exerts significant therapeutic effects in alleviating symptoms associated with T2DM, effects that are highly likely mediated through the modulation of gut microecological balance (9, 10). Gut microecological homeostasis primarily depends on the coordinated regulation of four core dimensions: the structure and composition of the gut microbiota, the production of gut-derived metabolites, the integrity of the intestinal barrier, and the dynamic balance of inflammation. Studies have shown that acupuncture intervention promotes the proliferation of beneficial bacteria while inhibiting the expansion of pathogenic species, thereby effectively ameliorating the dysbiotic state commonly observed in patients with T2DM (11). Gut microbiota dysregulation plays a critical role in the onset and progression of T2DM, characterized by imbalances in microbial community structure, aberrant gut-derived metabolites, and impaired intestinal barrier function (12, 13). These abnormalities collectively drive chronic low-grade inflammation, exacerbate insulin resistance, and impair pancreatic β-cell function, ultimately contributing to the development of T2DM. Focusing on the coordinated regulation of the functional dimensions of gut microecology represents a crucial research direction for elucidating the molecular mechanisms underlying acupuncture treatment of T2DM.

**Figure 1:**
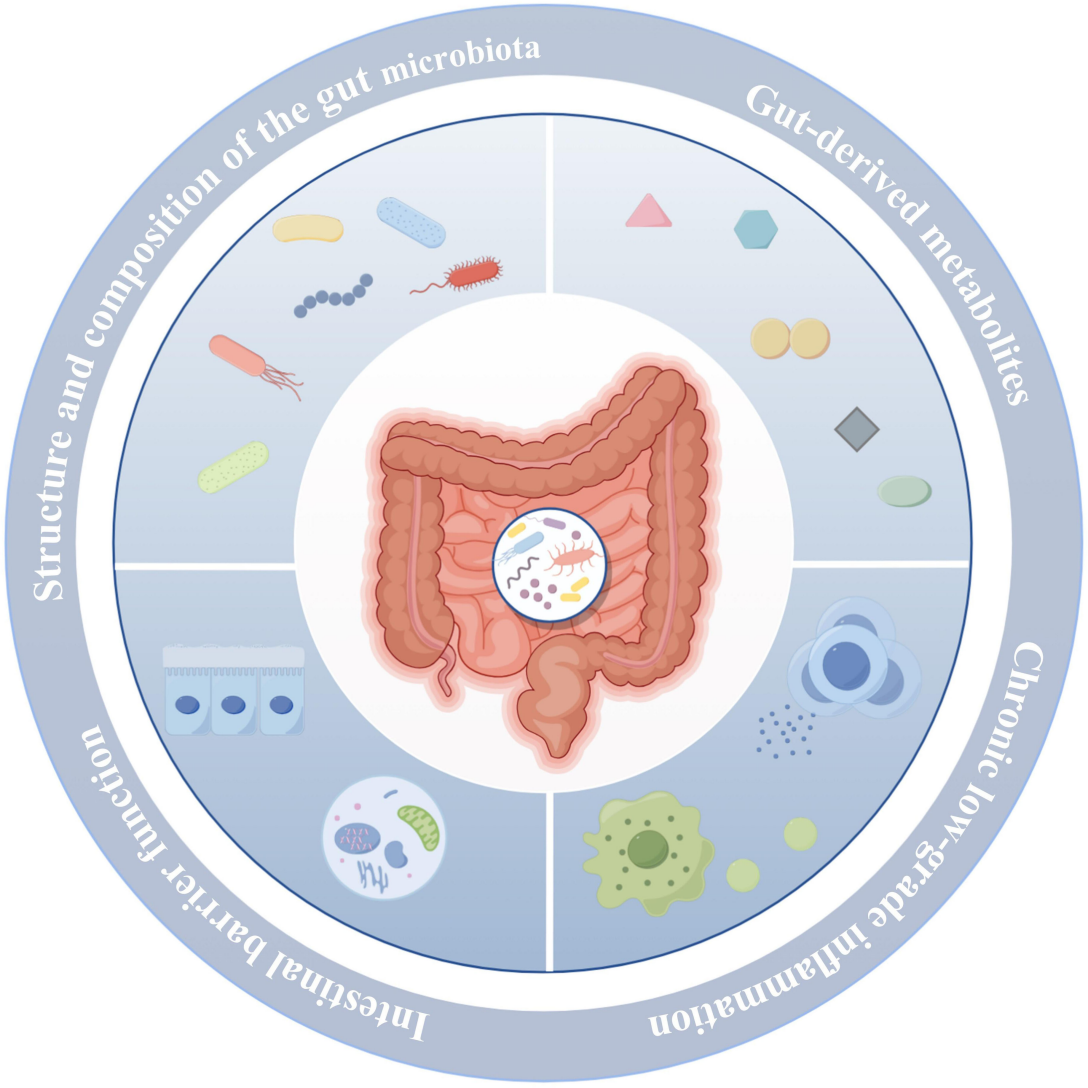
Four key functional dimensions of gut microbiota homeostasis. The figure was created using Figdraw 2.0.

Current research has made preliminary progress in elucidating the role of acupuncture in modulating gut microecology to intervene in T2DM; however, its precise underlying mechanisms remain incompletely understood. The core components of the gut microbiota are depicted in **Figure 1**. This review aims to systematically delineate the critical roles of gut microbiota dysbiosis, aberrant gut-derived metabolites, intestinal barrier dysfunction, and chronic low-grade inflammation in the pathogenesis of T2DM. It focuses on summarizing the potential mechanisms by which acupuncture ameliorates T2DM through the modulation of gut microecological homeostasis, and further explores the prospects and challenges of its application in individualized, precision treatment of T2DM. Ultimately, this review seeks to provide a theoretical foundation and practical guidance to optimize basic research directions and facilitate clinical translation of acupuncture in the prevention and treatment of metabolic diseases.

## 2 Gut microecological dysregulation as a key contributor to the pathogenesis of T2DM

Gut microecological imbalance is widely recognized as a critical pathogenic factor in the onset of T2DM. It is characterized by a decline in beneficial bacteria, such as *Bifidobacterium* and *Akkermansia*, alongside an expansion of pathogenic species, including *Escherichia coli* and *Clostridium* spp., resulting in a disrupted microbial community structure and composition. This dysbiotic state leads to impaired synthesis and functional dysregulation of gut-derived metabolites, manifested by reduced production of short-chain fatty acids (SCFAs), elevated lipopolysaccharide (LPS) release, and aberrant secondary bile acid profiles. Microbial imbalance and aberrant gut-derived metabolites disrupt intestinal barrier function, initiating systemic chronic low-grade inflammation. This inflammatory state suppresses insulin secretion and impairs insulin sensitivity in target tissues, thereby exacerbating insulin deficiency and resistance—central pathological mechanisms driving the onset and progression of T2DM.

### 2.1 Imbalance of gut microbiota structure and composition

The gut microbiota constitutes a highly complex and dynamic micro-ecosystem composed of diverse phyla, including *Firmicutes*, *Bacteroidetes*, *Actinobacteria*, *Proteobacteria*, and *Verrucomicrobia*. It is involved in multiple physiological processes such as carbohydrate and lipid metabolism, maintenance of intestinal barrier integrity, immune modulation, and neural signaling. Collectively, the gut microbiota plays an indispensable role in preserving host health and metabolic homeostasis. Under healthy conditions, the gut microbiota maintains a relatively stable community structure characterized by diverse species and balanced abundance. Symbiotic interactions and metabolic cooperation among different microbial taxa collectively sustain this microecological equilibrium. However, in the context of T2DM, this balance is disrupted, leading to disturbances in the structure and composition of the microbiota.

**Figure 2:**
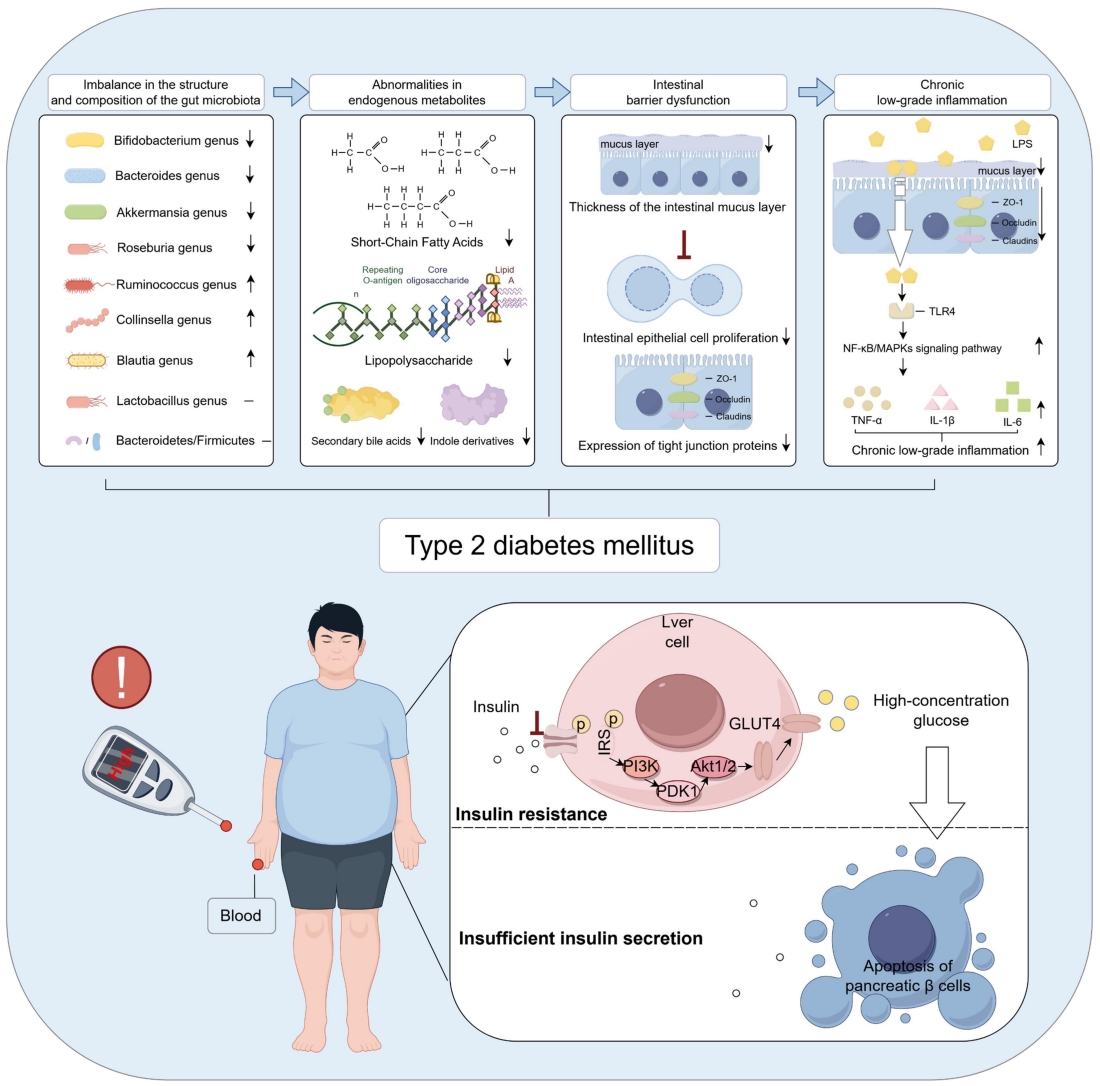
Gut microbiota dysbiosis is an important contributing factor in the pathogenesis of T2DM. The figure was created using Figdraw 2.0. TLR4: Toll-like receptor 4; TNF-α: Tumor necrosis factor-a; IL-1β: Interleukin-1β; IL-6: Interleukin-6; IRS: Insulin receptor substrate-1; PI3K: Phosphoinositide 3-Kinase; PDK1: 3-Phosphoinositide-Dependent Protein Kinase-1; GLUT4: Glucose Transporter Type 4.

Multiple independent studies have consistently reported a reduced relative abundance of genera such as *Bifidobacterium*, *Bacteroides*, *Akkermansia*, and *Roseburia* in the gut microbiota of T2DM patients, suggesting their potential protective roles in maintaining host metabolic homeostasis. Conversely, genera including *Ruminococcus*, *Collinsella*, and *Blautia* are frequently enriched in T2DM-associated microbiomes, implicating them as possible contributors to pro-inflammatory or pathogenic processes in the onset and progression of T2DM. Notably, the relative abundance of *Lactobacillus* exhibits inconsistent trends across T2DM studies, a variability that may reflect differences in strain diversity, host heterogeneity, intervention parameters, and sample origin. Moreover, although the Bacteroidetes-to-Firmicutes ratio has long been considered a potential microecological marker of metabolic diseases, current studies examining its association with T2DM yield inconsistent results, lacking robust and reproducible evidence. This variability suggests that reliance on broad taxonomic ratios alone may be insufficient to accurately capture the complexity of microecological dysbiosis associated with T2DM (**Table 1**). A visual representation of the content discussed in this subsection is provided in **Figure 2**.

**Table 1:** Gut microbiota characteristics in T2DM patients.

Disease	Study grouping	Sample collection and testing methods	Structure and composition of the gut microbiota (T2DM patients)	Ref.
T2DM	N=13, healthy controls N=40, T2DM patients	Stools 16S rRNA and metagenomic sequencing	↓: the shannon diversity index. ↓: the relative abundance of *Bacteroides*, *Prevotella*, *Lachnospira*, *Roseburia* and *Faecalibacterium*; ↑: the relative abundance of *Enterobacteriaceae*, *Collinsella*, *Streptococcus*, *Lactobacillus*.	(14)
T2DM	N=18, healthy individuals N=18, T2DM patients	Faecal samples 16S rRNA sequencing	↓: the relative abundance of *Bifidobacterium*; ↑: the relative abundance of *Lactobacillus*, *Fusobacterium*.	(15)
T2DM	N=12, healthy individuals N=16, T2DM patients	Faecal samples qPCR	No significant difference in diversity between the two groups. ↓: the relative abundance of *Bacteroides vulgatus* and *Bifidobacterium*; ↑: the relative abundance of *Clostridium leptum* subgroup.	(16)
T2DM	N=12, healthy individuals N=16, T2DM patients	Faecal samples 16S rRNA sequencing	↓: the proportion of *Firmicutes*; ↑: the proportion of *Bacteroidetes* and *Proteobacteria*.	(17)
T2DM	N=52, lean individuals N=56, abdominally obese patients with T2DM	Faecal samples Metagenomic sequencing	No significant differences were observed at the *Firmicutes*, *Bacteroidetes* phylum level among the two groups; ↓: the relative abundance of *Akkermansia muciniphila*;	(18)
T2DM	N=43, normal glucose tolerance individuals N=53, T2DM patients	Faecal samples Metagenomic sequencing	↓: the relative abundance of *Lactobacillus*.	(19)
T2DM	N=23, healthy individuals N=23, T2DM patients	Faecal samples 16S rRNA sequencing	↓: the relative abundance of *Bacteroides*; ↑: the relative abundance of *Ruminococcus*.	(20)
T2DM	N=48, normal glucose tolerance individuals N=20, T2DM patients	Faecal samples 16S rRNA sequencing	↑: the relative abundance of *Blautia*.	(21)

“↓”represents a decrease or reduction; “↑”represents an increase or elevation.

### 2.2 Aberrant gut-derived metabolites

Gut-derived metabolites refer to a class of bioactive low-molecular-weight compounds produced by the gut microbiota through the metabolism of dietary components, host endogenous substances such as bile acids and amino acids, or their own physiological activities. These metabolites are extensively involved in regulating diverse physiological processes, particularly carbohydrate and lipid metabolism. Gut-derived metabolites primarily include SCFAs, LPS, secondary bile acids, and indole derivatives. Gut microbiota dysbiosis can markedly alter the composition, abundance, and biological activity of these metabolites, manifested as follows (1): Reduced SCFA levels. Studies have shown that gut microbiota dysbiosis results in a significant decrease in SCFAs—namely acetate, propionate, and butyrate (22, 23)—primarily due to the diminished abundance of SCFA-producing bacteria such as *Bifidobacterium*, *Akkermansia muciniphila*, *Bacteroides*, and butyrate-producing bacteria in T2DM patients (24, 25) (2). Elevated LPS levels. Microbial dysbiosis leads to an increased relative abundance of Gram-negative bacteria in the gut, driving enhanced synthesis and release of LPS (26, 27). This exacerbates systemic chronic low-grade inflammation, thereby further promoting insulin resistance (3). Reduced levels of secondary bile acids. Gut microbiota dysbiosis leads to a decreased abundance of bacteria with dehydroxylation capabilities, such as *Clostridium* species (28), thereby impairing the biotransformation of primary to secondary bile acids. This disruption attenuates farnesoid X receptor (FXR) and TGR5-mediated signaling pathways, compromising glucose homeostasis and insulin sensitivity, and subsequently exacerbating dysregulated carbohydrate and lipid metabolism (4). Decreased levels of indole derivatives. Microbial dysbiosis also disrupts the metabolism of dietary tryptophan by gut microbes, resulting in reduced production of indolepropionic acid–a tryptophan metabolite known to enhance insulin secretion from pancreatic β-cells. Studies have demonstrated that circulating indolepropionic acid levels are significantly lower in individuals with T2DM compared to healthy controls (29). In summary, gut microbiota dysbiosis orchestrates the generation and regulation of multiple gut-derived metabolites, collectively driving dysregulated carbohydrate and lipid metabolism as well as chronic inflammation. This interplay provides a fundamental pathophysiological basis for the development of T2DM. A visual representation of the content discussed in this subsection is provided in **Figure 2**.

### 2.3 Intestinal barrier dysfunction

Gut microbiota dysbiosis and aberrant levels of gut-derived metabolites, particularly SCFAs, further exacerbate intestinal barrier dysfunction. The intestinal barrier serves as a critical physical and immunological interface between the internal and external environments. It is primarily composed of the gut microbiota, mucus layer, intestinal epithelial cells, and their tight junction proteins (30). This barrier performs multiple physiological functions, including preventing the translocation of harmful microbes and their metabolites across the intestinal wall (31), maintaining gut microecological balance, and regulating both local and systemic immune responses (32). Studies have shown that gut microbiota dysbiosis leads to a significant reduction in mucus layer thickness (33, 34). This alteration is primarily attributed to a decline in butyrate-producing bacteria, resulting in decreased synthesis of SCFAs–particularly butyrate–which suppresses goblet cell differentiation and the expression of the mucin MUC2 (35), thereby impairing mucus layer formation. Concurrently, overgrowth of mucus-degrading bacteria such as *Bacteroides*, along with inflammation-induced disruption of mucus secretion, further compromises the structural integrity and function of the mucus barrier, ultimately weakening the intestinal mucosa’s defense against pathogenic microbes and harmful metabolites. Furthermore, gut microbiota dysbiosis and aberrant gut-derived metabolites markedly inhibit the proliferation and renewal of intestinal epithelial cells (36, 37), while downregulating the expression of tight junction proteins such as ZO-1, occludin, and claudins (38, 39). This disruption compromises the epithelial barrier structure, increases intestinal permeability, and leads to barrier dysfunction. As a result, harmful bacterial metabolites like LPS and inflammatory mediators readily translocate into the lamina propria and systemic circulation, triggering immune activation and chronic low-grade inflammation, which further exacerbate metabolic dysregulation and insulin resistance. A visual representation of the content discussed in this subsection is provided in **Figure 2**.

### 2.4 Chronic low-grade inflammation

Chronic low-grade inflammation is widely recognized as a critical pathological feature in the onset and progression of T2DM. A central pathogenic mechanism involves gut microbiota dysbiosis, which elevates levels of LPS and impairs intestinal barrier function, thereby facilitating the translocation of LPS across the gut mucosal barrier into the systemic circulation. Circulating LPS binds to Toll-like receptor 4 (TLR4), activating canonical inflammatory signaling pathways including TLR4/MyD88/NF-κB and mitogen-activated protein kinases (MAPKs). This activation induces the excessive release of pro-inflammatory cytokines such as tumor necrosis factor-α (TNF-α), interleukin-1β (IL-1β), and interleukin-6 (IL-6) (40, 41), thereby eliciting persistent low-grade inflammation across multiple target tissues. The resulting chronic inflammation activates serine kinases, including JNK and IKK, which phosphorylate the insulin receptor and impair its normal binding to insulin (42), culminating in insulin resistance. This mechanism profoundly compromises insulin-mediated glucose uptake, markedly attenuating the responsiveness of key target tissues such as skeletal muscle, liver, and adipose tissue. Furthermore, the sustained hyperglycemia consequent to insulin resistance intensifies metabolic stress within pancreatic β-cells, precipitating a cascade of deleterious events including oxidative stress, chronic inflammation, and endoplasmic reticulum stress. Collectively, these insults drive β-cell apoptosis (43–45), thereby accelerating the pathogenesis of T2DM. A visual representation of the content discussed in this subsection is provided in **Figure 2**.

## 3 Mechanisms underlying acupuncture therapy for T2DM

### 3.1 Mechanisms of acupuncture in improving T2DM via regulation of gut microecological balance

Acupuncture, as a longstanding, safe, and minimally invasive traditional intervention, has increasingly demonstrated unique advantages in the prevention and treatment of metabolic diseases. By modulating the gut microecology through multi-target and multi-pathway mechanisms, acupuncture effectively ameliorates T2DM. Its primary actions include regulating gut microbiota composition and diversity, restoring gut-derived metabolite profiles, enhancing intestinal barrier integrity, and suppressing chronic low-grade inflammation.

**Figure 3:**
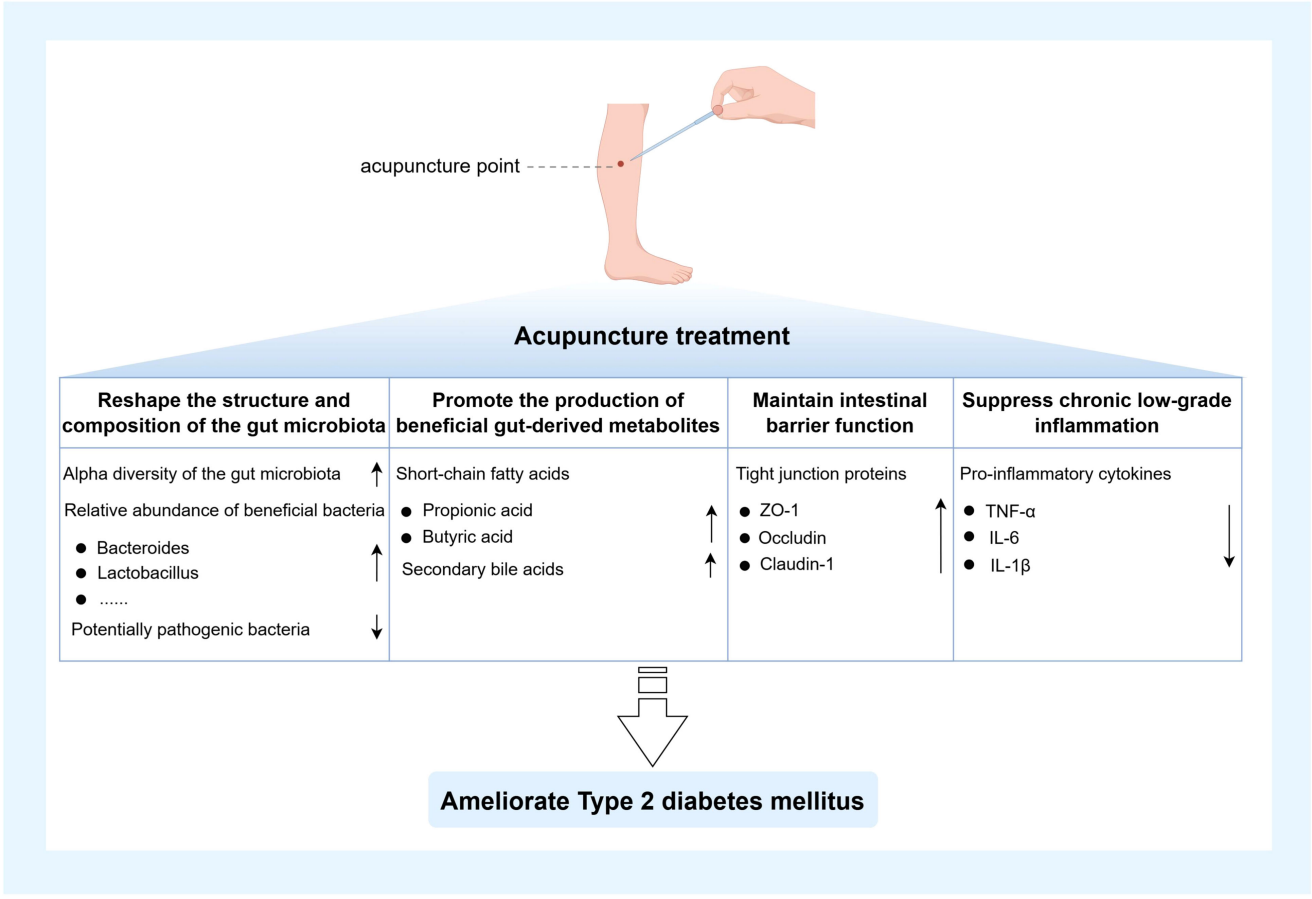
Mechanisms of acupuncture alleviating T2DM by modulating gut microbiota homeostasis. The figure was created using Figdraw 2.0. TNF-α: Tumor necrosis factor-α; IL-1β: Interleukin-1β; IL-6: Interleukin-6.

First, acupuncture markedly ameliorated gut microbiota dysbiosis associated with T2DM. Evidence from animal studies demonstrated that acupuncture intervention significantly enhanced gut microbial α-diversity and notably increased the relative abundance of key probiotics, including *Lactobacillus* and *Bacteroides* (10). These bacterial taxa can exert anti-inflammatory effects through the production of SCFAs, which reduce the generation of IKK and subsequently inhibit its interaction with the insulin receptor, thereby alleviating insulin resistance. In addition, SCFAs and their associated signaling pathways can promote the secretion of glucagon-like peptide-1 (GLP-1), which is closely linked to pancreatic β-cell proliferation and insulin secretion (46, 47). Corresponding clinical investigations revealed that acupuncture treatment induced substantial remodeling of the gut microbiota in T2DM patients, characterized by enrichment of beneficial microbes and reduction of potential pathogens. This ecological optimization suggested that acupuncture may indirectly modulate host glucose metabolism and insulin sensitivity by restoring microbial homeostasis (48). A study employing a T2DM mouse model revealed that electroacupuncture markedly reduced the relative abundance of diabetes-associated bacterial taxa, including *Lachnoclostridium*, *Lachnospiraceae_UCG-006*, *Odoribacter*, and *Oscillibacter*. Further fecal microbiota transplantation experiments demonstrated that the gut microbiota derived from electroacupuncture-treated mice significantly improved blood glucose levels and insulin resistance in antibiotic-treated diabetic mice (49). Moreover, when type 2 diabetic mice were co-treated with electroacupuncture and antibiotics, the hypoglycemic effect of electroacupuncture was abolished (50), suggesting that the gut microbiota plays a pivotal role in mediating the antidiabetic effects of electroacupuncture. Secondly, the role of acupuncture in modulating gut-derived metabolites has gained increasing attention. Multiple studies have demonstrated that stimulation of specific acupoints can influence microbial metabolic activity and promote the production of key metabolites. Acupuncture at “Zusanli” (ST36) and “Sanyinjiao” (SP6) has been shown to significantly elevate SCFA levels, particularly butyrate and propionate, thereby improving insulin sensitivity and enhancing glucose metabolic capacity (51). Similarly, acupuncture at “Pishu” (BL20) and “Weishu” (BL21) has also been reported to upregulate SCFA production, wherein butyrate activates the gut-expressed G protein-coupled receptor 43, thereby promoting insulin secretion and alleviating glucose metabolic disturbances (52). In addition to modulating SCFA production, acupuncture at “Tianshu” (ST25) and “Zusanli” (ST36) has been shown to enhance the synthesis of secondary bile acids, which, through activation of the FXR and TGR5 signaling pathways along the gut–liver axis, improve hepatic insulin sensitivity and facilitate glucose metabolism (53). Notably, FXR activation also suppresses hepatic cholesterol accumulation, thereby contributing to the amelioration of T2DM-associated lipid metabolic disturbances (54). Moreover, acupuncture has demonstrated pronounced biological effects in preserving intestinal barrier integrity. Multiple studies have shown that stimulation at “Tianshu” (ST25), “Zusanli” (ST36), “Zhongwan” (CV12), and “Pishu” (BL20) markedly upregulated the expression of key tight junction proteins in intestinal epithelial cells, including ZO-1, occludin, and claudin-1, thereby reinforcing mucosal barrier stability, reducing intestinal permeability, and restricting the translocation of luminal endotoxins such as LPS into the systemic circulation (52, 55). This barrier-protective effect helps attenuate systemic inflammation and interrupts the progression of insulin resistance driven by barrier dysfunction. Acupuncture also plays a pivotal role in suppressing chronic low-grade inflammation associated with T2DM. In chronic inflammation models driven by gut microbiota dysbiosis, acupuncture intervention effectively downregulated the expression of pro-inflammatory cytokines, including TNF-α, IL-6, and IL-1β (56). Further investigations revealed that stimulation at “Guanyuan” (CV4) and “Shenshu” (BL23) mitigated systemic inflammatory responses by inhibiting the activation of inflammatory signaling pathways such as NF-κB, thereby ameliorating insulin resistance and slowing the pathological progression of T2DM (57). In summary, by orchestrating an integrated modulation of the critical “gut microbiota–metabolites–intestinal barrier–inflammation” regulatory axis, acupuncture effectively alleviates insulin resistance driven by gut microecological dysbiosis and holds promise as a valuable therapeutic strategy for targeting both T2DM and gut microecological dysbiosis. A visual representation of the content discussed in this subsection is provided in **Figure 3**.

### 3.2 Direct mechanisms of acupuncture in ameliorating T2DM

Beyond indirectly improving glucose metabolism through modulation of the gut microecological balance, acupuncture also directly targets multiple pivotal pathways in the treatment of T2DM. It effectively modulates relevant signaling pathways involved in glucose metabolism. Studies have demonstrated that electroacupuncture at Zusanli (ST36) and Shenshu (BL23) reduces peripheral insulin levels while significantly upregulating hepatic mRNA expression of GLUT2 and glucokinase (GCK) (58), thereby enhancing hepatic glucose uptake and utilization, ultimately ameliorating hyperglycemia. Furthermore, acupuncture modulates the serine and tyrosine phosphorylation of insulin receptor substrate-1 (IRS-1) in skeletal muscle, upregulating the expression of IRS-1, IRS-2, and GLUT4. This enhances insulin signaling pathway activity and effectively reduces peripheral insulin resistance (59). Acupuncture also exhibits significant direct regulatory effects on pancreatic islet function. By activating the local pancreatic neural network, acupuncture promotes structural repair of the pancreas, enhances both the secretory capacity and mass of islet β-cells, and stimulates insulin secretion. Studies suggest that acupuncture may activate the PDX-1/GLUT2/GCK signaling pathway and upregulate gut-derived GLP-1 expression, thereby promoting β-cell proliferation and preserving their functional integrity (60, 61). Meanwhile, acupuncture also possesses anti-apoptotic properties toward pancreatic β-cells. Studies examining the effects of electroacupuncture on circRNA expression in plasma exosomes revealed that it inhibits β-cell apoptosis by modulating thyroid hormone and phosphatidylinositol signaling pathways (62). Additionally, acupuncture has been shown to markedly reduce aberrant mitochondrial cytochrome c release, suppress the activation of key pro-apoptotic factors such as caspase-9 and caspase-3, and thereby block the mitochondrial-mediated apoptotic cascade, preserving β-cell functional stability and viability (63, 64).

In summary, acupuncture not only indirectly modulates the progression of T2DM through regulation of the gut microecology but also directly targets glucose metabolic pathways, islet function, and cellular proliferation and apoptosis. This multi-level, multi-target systemic intervention provides a robust theoretical foundation for acupuncture as a non-pharmacological therapeutic strategy for T2DM.

## 4 Prospects and challenges of acupuncture modulation of gut microecology for T2DM treatment

### 4.1 Prospects

The application prospects of acupuncture in modulating gut microecology for the treatment of T2DM have garnered increasing attention (65). As a traditional medicine–derived, safe, and well-tolerated non-pharmacological intervention, acupuncture demonstrates multi-pathway and multi-target systemic regulatory capabilities. Acupuncture can play a positive role in regulating insulin resistance and improving abnormal glucose and lipid metabolism by reshaping gut microbiota composition and structure (66), promoting the synthesis of advantageous gut-derived metabolites (67), enhancing intestinal barrier integrity (68) and suppressing chronic low-grade inflammation (69). This not only provides a biological foundation for acupuncture’s therapeutic role in T2DM but also supports its development as a novel strategy for managing metabolic diseases. Secondly, driven by the principles of precision medicine, acupuncture interventions hold promise for integration with individualized gut microecological profiles, enabling a targeted therapeutic paradigm of “microbial typing–targeted acupoint selection–individual response prediction.” With advancing insights into the associations between gut microbial subtypes and metabolic phenotypes (70), the development of stratified intervention models based on microbiota characteristics is emerging as a key direction for future research. Furthermore, owing to its ease of administration and cost-effectiveness, acupuncture presents substantial potential for widespread implementation, particularly among T2DM patients with multiple metabolic comorbidities or those at risk of adverse drug reactions. Its adaptability across primary healthcare settings, rehabilitation centers, and community health management facilitates the establishment of a multi-tiered, sustainable integrated intervention framework for T2DM. With ongoing advances in mechanistic research, technological innovation, and the accumulation of robust clinical evidence, acupuncture-mediated modulation of gut microecology is poised to become a pivotal component of precision treatment strategies for T2DM, offering a model pathway for the modernization and global integration of traditional Chinese medicine.

### 4.2 Challenges

Despite demonstrating promising efficacy in clinical studies for the treatment of T2DM and other metabolic disorders, acupuncture research and application remain constrained by numerous limitations, which significantly hinder its broader adoption and deeper integration. The following provides an overview of the key challenges and issues currently confronting acupuncture interventions for T2DM.

#### 4.2.1 Uncertainties in the mechanisms of acupuncture modulation of the autonomic nervous system

Acupuncture, as a therapeutic modality that modulates physiological functions through stimulation of specific acupoints, is generally believed to exert its effects via the autonomic nervous system (71). The autonomic nervous system, particularly the vagus and sympathetic nerves, plays a pivotal role in regulating metabolic diseases (72). Acupuncture influences visceral organ function by activating the vagus nerve, thereby ameliorating metabolic disorders such as diabetes. However, the precise mechanisms by which acupuncture modulates the autonomic nervous system remain incompletely understood. Specifically, how acupuncture activates the vagus nerve through stimulation of distinct acupoints and subsequently modulates endocrine and metabolic processes remains underexplored in a systematic manner. Current studies primarily focus on the activation effects of acupuncture on the vagus nerve, yet the differential outcomes elicited by various acupoints, the precise neural pathways involved, and the crosstalk between the nervous, endocrine, and immune systems warrant deeper investigation. Furthermore, individual variability presents a significant challenge, as differential neural responses among subjects may contribute to the heterogeneity of acupuncture’s therapeutic efficacy, necessitating rigorous quantification and optimization in future research.

#### 4.2.2 Insufficiency of in-depth mechanistic validation

Despite emerging clinical evidence supporting acupuncture’s efficacy in treating metabolic diseases such as diabetes, the precise molecular mechanisms underlying its therapeutic effects remain insufficiently validated. Most existing studies have focused on acupuncture’s modulation of gut microecology, immune responses, and glucose-lipid metabolism to alleviate metabolic symptoms (11, 73). However, in-depth exploration of specific biomarkers, signaling pathways, and molecular mechanisms involved in acupuncture’s action is still markedly lacking. Although acupuncture is believed to improve diabetes metabolism by modulating gut microbial composition (74), the specific bacterial taxa or metabolic products mediating these effects remain unclear. Future investigations urgently require larger-scale animal studies, cellular experiments, and clinical trials to elucidate and validate the biological mechanisms of acupuncture, thereby providing a more robust theoretical foundation and clinical evidence.

### 4.3 Future research directions

Future research on acupuncture treatment for T2DM should leverage multi-omics integration to comprehensively investigate underlying mechanisms, establish standardized therapeutic protocols, and develop robust efficacy evaluation frameworks.

#### 4.3.1 Multi-omics integrative research

Multi-omics integrative research, as a pivotal frontier in modern biomedical science, offers a systematic and comprehensive framework to elucidate the mechanisms underlying acupuncture treatment for T2DM and other metabolic disorders. By integrating multidimensional datasets encompassing genomics, transcriptomics, metabolomics, and microbiomics, this approach enables a holistic dissection of acupuncture’s biological effects across gene expression regulation, metabolic alterations, and gut microecological remodeling. A comprehensive study employing single-cell sequencing, transcriptomics, metabolomics, and metagenomics systematically evaluated the effects of acupuncture in patients undergoing methadone maintenance therapy. The findings demonstrated that acupuncture exerts regulatory effects through multiple biological layers, including the modulation of immune function, reshaping of gut microbial composition and metabolic activity, and regulation of cellular immune signaling pathways. These results provide systematic and molecular-level evidence supporting the potential of acupuncture as an adjunctive therapy for mitigating the adverse effects associated with long-term opioid use (75). Integrative analyses based on multi-omics data not only facilitate the identification of key molecular pathways and targets mediating acupuncture’s therapeutic effects but also enable the elucidation of individualized response profiles. Moreover, by combining genomic information with gut microbiome characteristics, these approaches can assist in predicting treatment outcomes and guide the development of personalized acupuncture regimens. With ongoing advancements in multi-omics technologies and continual refinement of data analytical methods, future studies are poised to further unravel the complex network mechanisms underlying acupuncture’s modulation of metabolic diseases and accelerate its translation and application within the realm of precision medicine.

#### 4.3.2 Establishing standardized acupuncture protocols and evaluation frameworks

Standardization of acupuncture therapy remains a significant challenge in current research. Considerable individual variability in acupuncture treatment–including differences in acupoint selection, stimulation intensity, treatment frequency, and duration–has led to poor comparability and reproducibility across studies. Therefore, establishing unified, standardized treatment protocols and evaluation frameworks is essential to enhance the scientific rigor and clinical applicability of acupuncture. Firstly, standardization requires clear definition of key parameters such as acupoint selection, stimulation intensity, treatment frequency, and course duration. Existing studies have predominantly focused on traditional and commonly used acupoints, such as Zusanli (ST36) and Tianshu (ST25) (76–78), yet symptomatology, disease severity, and individual patient differences may necessitate more precise acupoint targeting. Future research could integrate artificial intelligence and big data technologies to tailor personalized acupuncture protocols based on patients’ clinical characteristics and biomarkers. In studies aimed at standardizing key acupuncture parameters, the inclusion of appropriate control groups (e.g., sham acupuncture) is essential to ensure the scientific rigor and reproducibility of the findings. Secondly, standardization of evaluation systems is equally crucial. Currently, efficacy assessments of acupuncture largely rely on changes in clinical symptoms and routine biochemical markers, which are relatively limited and fail to comprehensively capture therapeutic outcomes. Moving forward, a multidimensional evaluation framework incorporating clinical symptoms, metabolic biomarkers, immune responses, and gut microbiota alterations should be established to provide a more holistic and objective appraisal of acupuncture efficacy. Moreover, clinical trial data based on standardized evaluation criteria will offer robust support for the wider adoption of acupuncture and inform policy development.

## 5 Conclusion

T2DM, a global chronic metabolic disorder, is increasingly recognized to be closely associated with gut microecological imbalance. In recent years, the role of the gut microbiota in regulating glucose metabolism, maintaining insulin sensitivity, and mediating insulin resistance has emerged as a major focus of research. Acupuncture, as a traditional medicine intervention with systemic regulatory advantages and a non-pharmacological approach, has been demonstrated to act on multiple pathological pathways related to T2DM. These include remodeling gut microbial composition and diversity, enhancing the production of beneficial gut-derived metabolites such as SCFAs, strengthening intestinal epithelial barrier function, suppressing pro-inflammatory signaling pathways, and ameliorating systemic chronic inflammation. By modulating the critical axis of “gut microbiota–metabolites–intestinal barrier–inflammation”, acupuncture holds promise for upstream intervention in T2DM-associated glucose dysregulation, offering novel insights for comprehensive disease management.

However, the precise molecular mechanisms by which acupuncture modulates T2DM through the gut microecology remain incompletely understood, with a notable lack of systematic and reproducible mechanistic validation. Future research should fully integrate multi-omics technologies–including genomics, transcriptomics, metabolomics, and microbiomics–combined with bioinformatics approaches to elucidate the key signaling pathways and core targets of acupuncture intervention. Concurrently, there is an urgent need to establish standardized acupuncture treatment parameters encompassing acupoint selection, stimulation intensity, intervention duration, and clinical efficacy evaluation criteria. Such efforts will facilitate the standardization, mechanistic clarification, and clinical practicability of acupuncture in the precision management of T2DM, thereby broadening its translational potential in metabolic disease therapeutics.
